# Structure-Function Study of Mammalian Munc18-1 and *C. elegans* UNC-18 Implicates Domain 3b in the Regulation of Exocytosis

**DOI:** 10.1371/journal.pone.0017999

**Published:** 2011-03-21

**Authors:** Margaret E. Graham, Gerald R. Prescott, James R. Johnson, Mathew Jones, Alice Walmesley, Lee P. Haynes, Alan Morgan, Robert D. Burgoyne, Jeff W. Barclay

**Affiliations:** Department of Cellular and Molecular Physiology, The Physiological Laboratory, Institute of Translational Medicine, University of Liverpool, Liverpool, United Kingdom; The University of Queensland, Australia

## Abstract

Munc18-1 is an essential synaptic protein functioning during multiple stages of the exocytotic process including vesicle recruitment, docking and fusion. These functions require a number of distinct syntaxin-dependent interactions; however, Munc18-1 also regulates vesicle fusion via syntaxin-independent interactions with other exocytotic proteins. Although the structural regions of the Munc18-1 protein involved in closed-conformation syntaxin binding have been thoroughly examined, regions of the protein involved in other interactions are poorly characterised. To investigate this we performed a random transposon mutagenesis, identifying domain 3b of Munc18-1 as a functionally important region of the protein. Transposon insertion in an exposed loop within this domain specifically disrupted Mint1 binding despite leaving affinity for closed conformation syntaxin and binding to the SNARE complex unaffected. The insertion mutation significantly reduced total amounts of exocytosis as measured by carbon fiber amperometry in chromaffin cells. Introduction of the equivalent mutation in UNC-18 in *Caenorhabditis elegans* also reduced neurotransmitter release as assessed by aldicarb sensitivity. Correlation between the two experimental methods for recording changes in the number of exocytotic events was verified using a previously identified gain of function Munc18-1 mutation E466K (increased exocytosis in chromaffin cells and aldicarb hypersensitivity of *C. elegans*). These data implicate a novel role for an exposed loop in domain 3b of Munc18-1 in transducing regulation of vesicle fusion independent of closed-conformation syntaxin binding.

## Introduction

Membrane fusion is driven by a precise series of protein-protein interactions that enable lipid bilayers to merge. Exocytosis requires the assembly of the SNARE (soluble N-ethylmaleimide sensitive factor attachment protein receptor) complex, which at mammalian synapses is comprised of syntaxin-1, VAMP (vesicle associated membrane protein) and SNAP-25 (synaptosomal associated protein of 25 kDa) [1]. Although SNARE proteins can be sufficient to drive membrane fusion on their own [2], the precise temporal and spatial restrictions on exocytosis implicate the requirement of additional factors. Sec1/Munc18-1 (SM) proteins are a family of essential proteins that function in multiple aspects of membrane fusion [3]–[5] via interactions with syntaxin either on its own or within the assembled SNARE complex.

Originally described as orthologues from genetic screens in *C. elegans* [6] or yeast [7] Munc18-1 is a 67 kDa protein primarily characterised via its tight interaction with closed conformation syntaxin [8]. The structural basis of this mode of interaction has been investigated in depth and involves both domain 1 and domain 3a of Munc18-1 [9]. A variety of mutations affecting this interaction have implicated it functionally in the recruitment/docking of vesicles [10], [11] upstream of the actual fusion reaction. In contrast, Munc18-1 can also bind to the extreme N-terminus of syntaxin [12]–[14] and this interaction appears to be more critical to the regulation of the fusion process itself [15]–[17], although this interpretation is not fully supported in other studies [18]. Munc18-1 can also interact with the assembled SNARE complex affecting vesicle priming and/or fusion [12], [15], [19], [20].

In addition to syntaxin, Munc18-1 can also interact with a number of accessory exocytotic proteins including Mint1 [21], granuphilin [22], Rab3 [10], Doc2 [23] and phospholipase D [24]. The functional implications of many of these interactions in regulated exocytosis remains poorly understood and the structural regions within the Munc18-1 protein important for these interactions are mostly unknown. In order to shed light on which regions of the Munc18-1 protein are involved in regulation of exocytosis independent of closed conformation syntaxin binding we undertook an unbiased structural-functional approach to insert 15 base pair linkers randomly into the Munc18-1 coding sequence. Assessment of their biochemical phenotype showed that, although many insertion mutants had syntaxin binding defects, one (Glu379) was specifically defective for Mint1 binding without any effect on the affinity for closed-conformation syntaxin or binding to the assembled SNARE complex. Glu379 is located in an exposed loop within domain 3b of Munc18-1, a region of the protein previously unspecified as being functionally important. We assessed the role of this mutation in exocytosis and found a reduction in neurotransmitter release in comparison to controls. From the effects of this mutation we suggest a novel role for domain 3b of Munc18-1 in regulating neuronal exocytosis.

## Results

Munc18-1 has been primarily characterised via its interaction with the SNARE protein syntaxin-1A via three distinct modes of binding; however, Munc18-1 can also modify regulated exocytosis through interactions with other proteins. Although a great deal is known regarding syntaxin binding [9], [12], [14], [15], [25] the structural regions of the Munc18-1 protein required for syntaxin-independent interactions are relatively uncharacterised. To that end we used an *in vitro* transposon system to insert 5 amino acids randomly throughout the protein, selected those mutations that did not truncate the protein and assessed the ability of the mutants to bind to two described binding partners, syntaxin-1 and Mint. Segments of protein sequence that are structurally inaccessible or part of an active site are generally intolerant of such small insertions whereas segments located on the outer surface of the protein or in connector regions are more readily accepted [26], [27]. Consequently mapping of the position of the mutation within the gene can enable analysis of protein regions or amino acids that are important in mediating Munc18-1 function.

**Figure 1:**
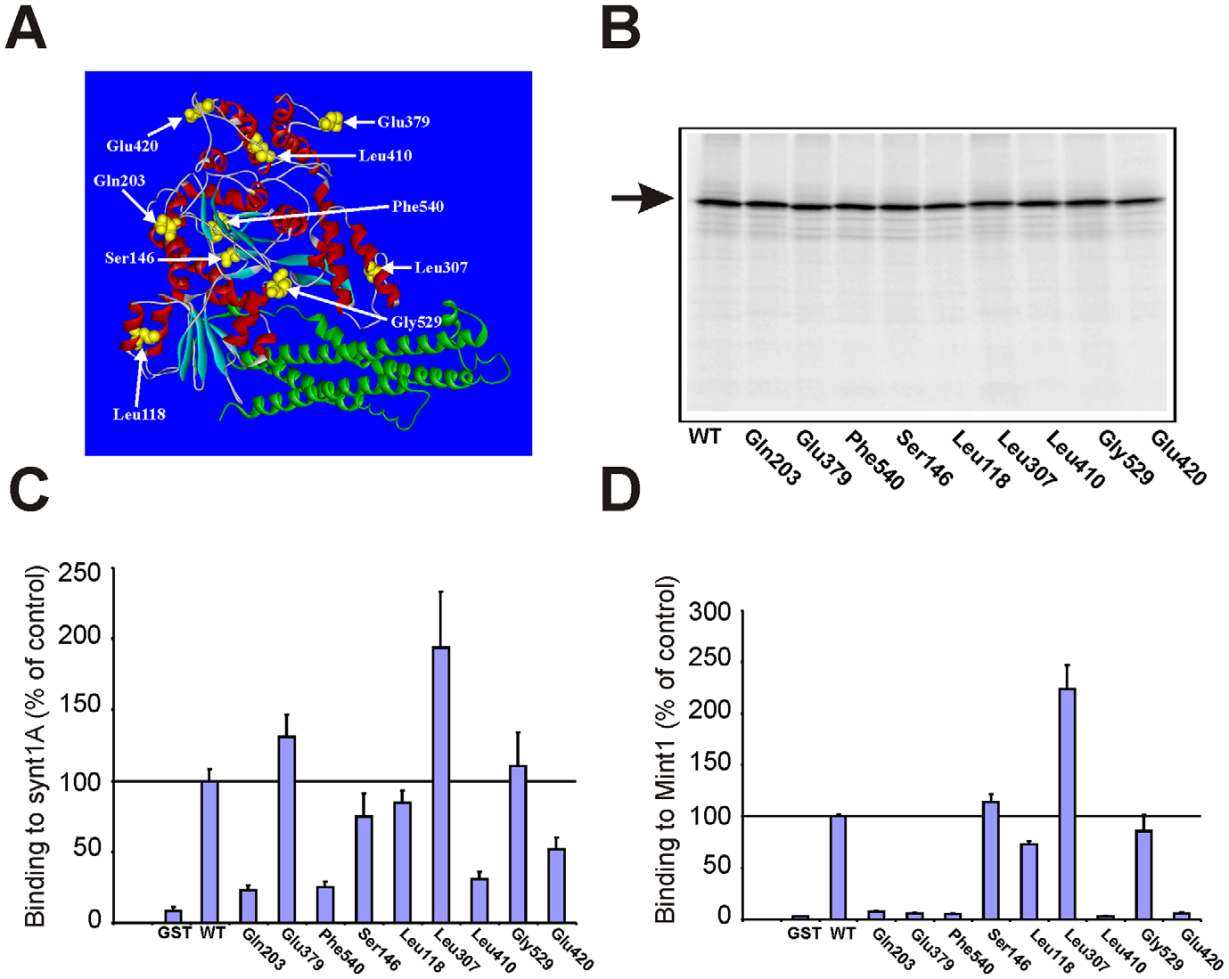
Biochemical phenotype of transposon insertion mutants of Munc18-1. (A) Structure of Munc18-1 indicating the position of the insertion mutations. Nine distinct full-length mutants were created randomly distributed throughout the Munc18-1 protein structure. For each mutant, five amino acids were inserted at the specified location (for the amino acid sequence of each exact insertion see Table 1). Arrows designate the position of the last unchanged amino acid for each insertion mutation. (B) ^35^S-radiolabelled Munc18-1 insertion mutants were produced by an *in vitro* transcription-translation reaction and separated by SDS-PAGE. Amount of protein made for wildtype (Wt) and mutant Munc18-1 were verified by autoradiography as indicated by the arrow. Note that none of these insertion mutations affected protein expression. Radiolabelled Munc18-1 proteins from part B were then assessed for binding to GST-syntaxin (C) and GST-Mint1-MBC (Munc18 binding domain) (D) and compared as a percentage of wildtype (Wt) binding. Despite equivalent levels of protein input the insertion mutations had variable effects on binding to syntaxin and Mint1. Glu379 mutant was of particular interest as it had no effect on syntaxin binding but completely eliminated binding to Mint1. The Ser146 mutant had no effect on binding to either protein.

Using the GPS-LS transposon system we inserted 15 base pairs randomly throughout the coding sequence of Munc18-1 (Figure 1A; Table 1). Expression of full-length Munc18-1 protein was verified by an *in vitro* transcription-translation reaction and it was found that none of the insertions had any gross effect on protein expression levels and that all products were of a similar size (Figure 1B). Radiolabelled proteins were then incubated in a glutathione-coated 96 well plate to which GST-syntaxin (residues 4-266 allowing closed-conformation binding only) had been previously attached. Bound Munc18-1 protein was then eluted, quantified and expressed as a percentage of wildtype binding. Producing Munc18-1 in this way produces approximately 5 pM radiolabelled protein which is far below the K_D_ for the syntaxin-1 interaction [28] and thus any potential reductions in binding affinities might be detectable with this assay. GST control wells were recorded as approximately 8–10% of wildtype value (Figure 1C); therefore, background counts were considered to be 10% of the positive control giving a 10-fold signal to noise ratio. Analysis of the various Munc18-1 insertion mutants revealed significant variation in syntaxin binding efficiency (Figure 1C). Mutants with insertions at Leu118, Ser146, Leu307, Glu379 and Gly529 bound syntaxin with similar efficiency to wildtype with no significant variation occurring between the values obtained. This result was not surprising as the binding surface for Munc18-1 for closed conformation syntaxin is primarily comprised of amino acids of domain 1 of Munc18-1 [9]. Mutations that have been reported to affect this mode of binding include Asp34 [29], Met38 [11] and Arg39 [30]. Surprisingly the Gln203, Leu410 and Phe540 insertion mutations all showed a significantly impaired ability to bind syntaxin (P<0.01) with values varying between 20–30% of wildtype binding (Figure 1C). In addition the Glu420 mutant had an approximately 50% reduction in affinity for syntaxin. The sites of these mutations within the protein structure have not been previously indicated as sites of interaction between Munc18-1 and syntaxin as these mutations are not contained within domain 1 or 3a.

**Table 1:** Munc18-1 insertion mutations as defined by the last unchanged amino acid, the orthologous residue in *Caenorhabditis elegans* and the exact 5 amino acid insertion.

Munc18-1amino acid	UNC-18amino acid	Insertion
Leu118	Leu116	VFKQL
Ser146	Asn144	CLNNS
Gln203	His201	LFKHQ
Leu307	Leu305	MFKHL
Glu379	Glu378	VFKHE
Leu410	Leu409	LFKHL
Glu420	Asp419	VFKHE
Gly529	Ser529	CLNTG
Phe540	Tyr540	CLNIF

Subsequent to syntaxin binding characterisation we tested the ability of the various insertion mutants to bind Mint1 by first immobilising the GST- tagged Munc18-1 binding domain (MBD) as bait protein and incubating with radiolabelled versions of the various mutant Munc18-1 proteins (Figure 1D). The MBD has been identified as the interaction site of Mint proteins for Munc18-1 [21]. Mint1 MBD was bound at the same molar concentration of syntaxin to allow for a direct comparison between the binding affinities for the two proteins. Similar to the Munc18-1/syntaxin experiment, Gln203, Leu410 and Phe540 all had a significantly reduced affinity of interaction with Mint1-MBD (Figure 1D). This may suggest that these insertions have disrupted whole protein conformation and consequently this would be likely to result in mutants with reduced binding affinity for any protein. The Glu420 mutant also showed binding reductions to both proteins, although binding to MBD was more severely affected in comparison to the 50% reduction seen for syntaxin. Insertions at Ser146 and Gly529 had no effect on binding to Mint1-MBD in comparison to wildtype protein (Figure 1D), as they also did for syntaxin binding. Leu118 had a moderate effect on Mint1 binding without an effect on syntaxin binding whereas Leu307 showed an enhanced interaction, binding with 225% of wildtype. Intriguingly the Glu379 mutant, which had no effect on syntaxin binding (Figure 1C), completely eliminated binding to Mint1 (Figure 1D).

**Figure 2:**
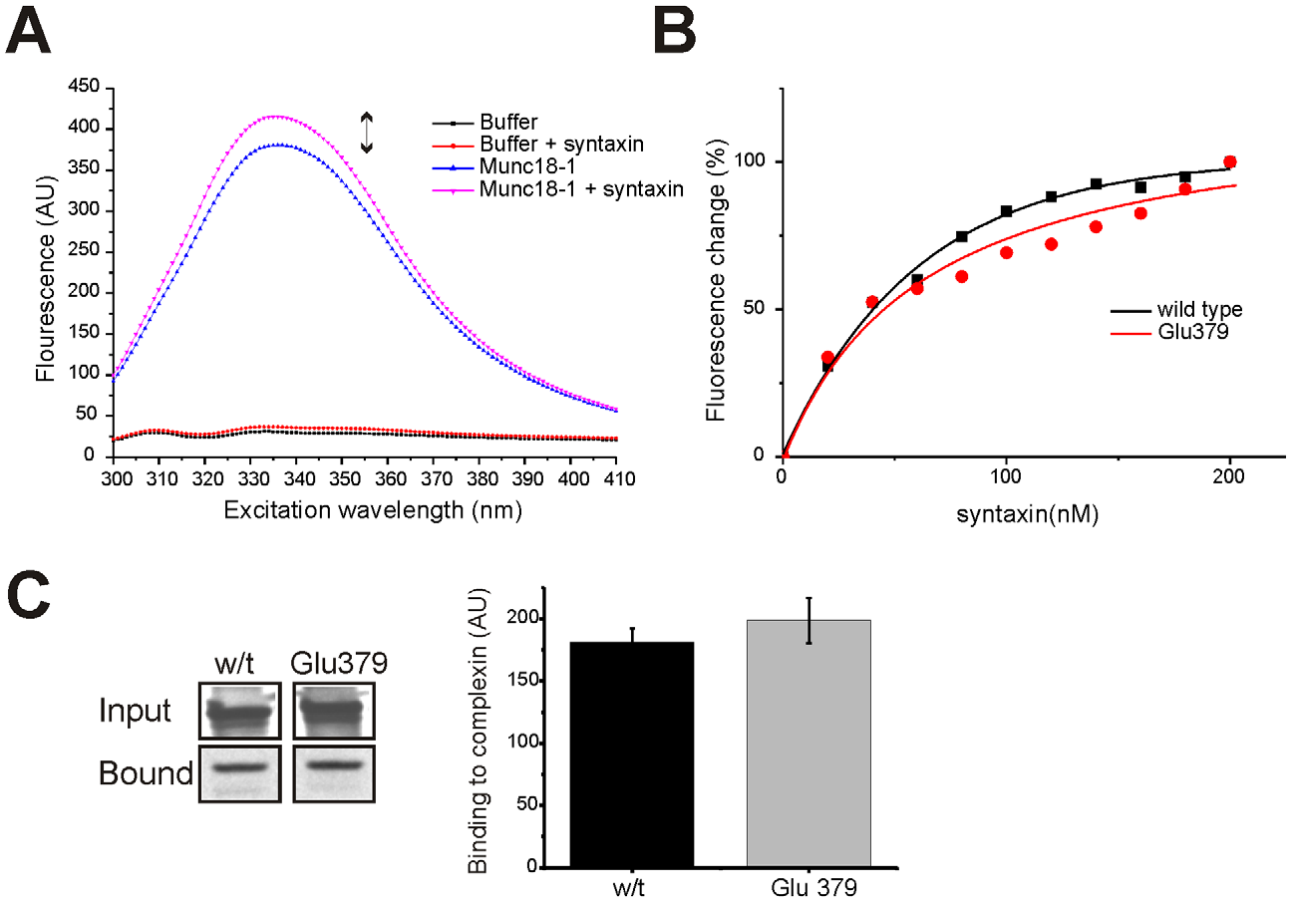
Lack of effect of the Glu379 mutation on the affinity of binding of Munc18-1 to closed syntaxin or on binding to the assembled SNARE complex. (A) Fluorescence emission spectra are shown of samples of buffer or buffer containing 100nM Munc18-1 with our without addition of 200 nM His_6_-syntaxin1A4-266. Addition of His_6_-syntaxin1A4-266 to Munc18-1 increased the peak fluorescence emission (indicated by the double-headed arrow). (B) Dose-dependent effect of addition of His_6_-syntaxin1A4-266 on peak fluorescence emission for wildtype and Glu379 Munc18-1. The data were fitted using non-linear curve fitting to a hyperbolic function. (C) A detergent-solubilized brain extract was incubated with GST-complexin immobilised on glutathione-Sepharose beads to pull down assembled SNARE complexes. In vitro translated, ^35^S-radiolabelled, Munc18-1 wildtype and Glu379 were incubated with the loaded glutathione-Sepharose beads and bound radiolabelled protein remaining after washing and the corresponding inputs were analysed by SDS-PAGE followed by exposure to ^35^S-sensitive film. The amount of bound radioactivity was assessed by quantitative densitometry (n = 3) of Munc18-1 wildtype and Glu379 binding to the GST-complexin affinity-purified neuronal SNARE complex and corrected for the level of input protein. No difference was seen in the level of binding of wildtype and mutant protein.

The Glu379 mutant showed no apparent defect in binding to closed syntaxin using the glutathione-coated plate assay but such an assay would not be at equilibrium due to washing of the plates prior to measurement of binding and so this assay would not necessarily detect changes in equilibrium binding affinity [31]. Previous work has determined the binding affinities of Munc18-1 for syntaxin using either isothermal calorimetry or by monitoring the change in fluorescence of the tryptophans present in Munc18-1 following binding of syntaxin [14] which does not itself possess a tryptophan. The two assays were found to give very similar results with a variety of constructs and we chose to use the fluorescence assay as it is a direct measure of changes in Munc18-1 conformation during and following the binding interaction. GST-fusion proteins for wild-type and Glu379 Munc18-1 were expressed, the GST removed by PreScission cleavage and tryptophan fluorescence of the free Munc18-1 protein monitored. Addition of His_6_-syntaxin1A4-266 to Munc18-1 elicited a clear increase in the peak fluorescence (Figure 2A) as described previously [14]. Upon titration of different amounts of His_6_-syntaxin1A4-266, a progressive increase in fluorescence was obtained over a similar concentration range as described previously [14]. There was little difference observed however in the apparent affinity for His_6_-syntaxin1A4-266 between wild-type and Glu379 Munc18-1 (Figure 2B). Since Munc18-1 is also able to bind in an alternative mode to the assembled SNARE complex [12], [13], [15], [32] we also tested the binding of Glu379 in an assay based on pull down of assembled SNARE complexes from a rat brain extract using GST-complexin. This assay was able to detect a reduction in the extent of SNARE complex binding of Munc18-1 with the mutation Y337L [33] in a domain that has recently been show to directly interact with SNARES [32]. In contrast, the Glu379 mutation had no effect on Munc18-1 binding to the immobilised SNARE complex in this assay (Figure 2C). Another mutation in Munc18-1 (E466K) has been found to act as a gain of function mutation that could reveal an interaction with Rab3 [10]. Munc18-1 with the Glu379 mutation did not show any evidence for increased Rab3 binding (data not shown).

**Figure 3:**
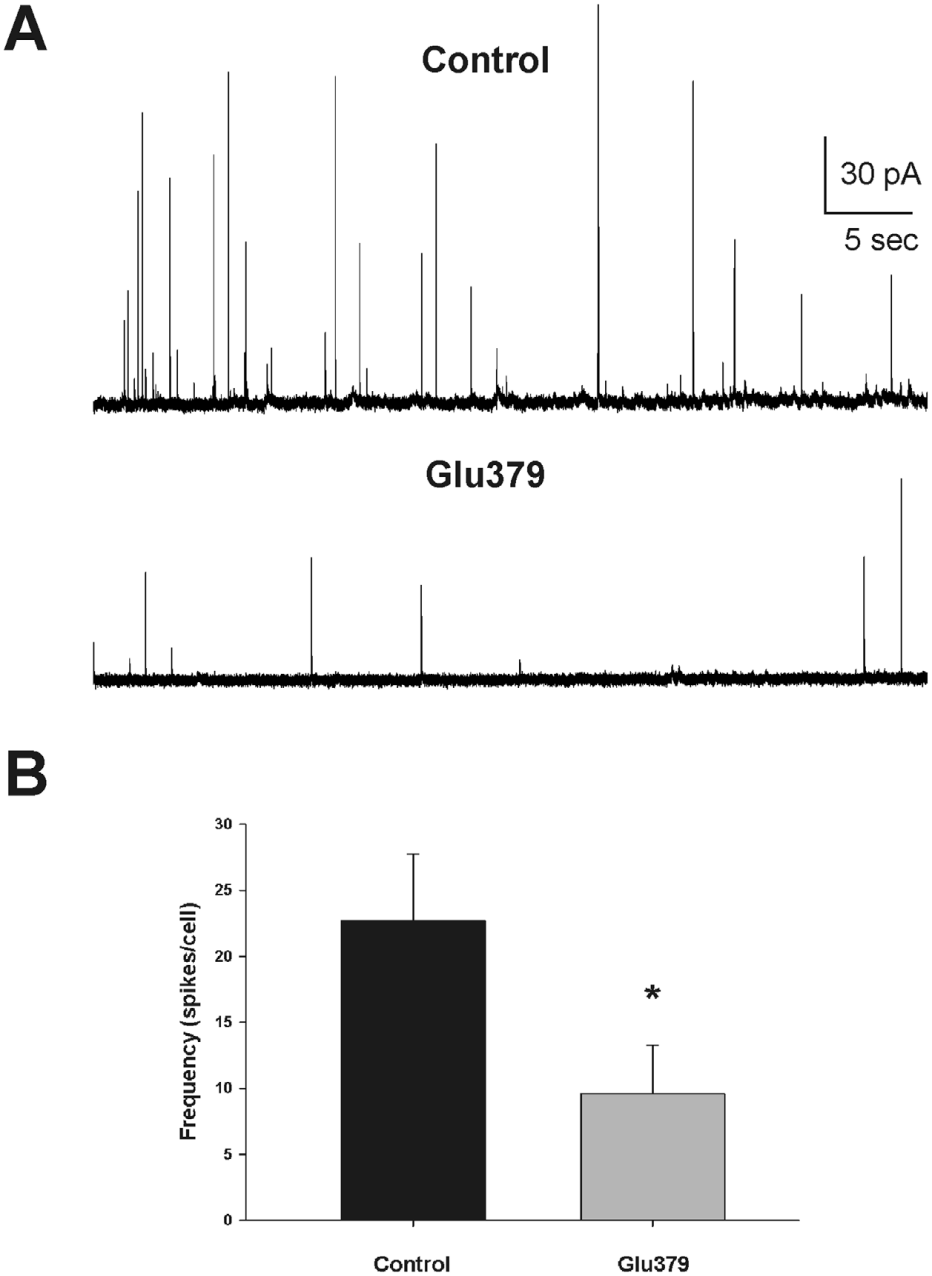
The Munc18-1 Glu379 insertion mutation reduces frequency of exocytosis. (A) Amperometric trace examples from bovine adrenal chromaffin cells expressing the Munc18-1 Glu379 insertion mutant (Glu379) or from untransfected cells (Control). (B) Frequency of individual fusion events was significantly reduced in Glu379 expressing cells in response to stimulation (*; p<0.01). Data were analysed from 431 spikes from 19 responding cells (control) and 67 spikes from 7 responding cells (Glu379). Note that the vast majority of Glu379 cells did not respond to stimulation at all.

Glu379 is located on the external surface of the Munc18-1 structure in an exposed loop near the beginning of domain 3b [9], a region of the protein that has no previously characterised role in protein binding. We hypothesized that this region may be important for transducing closed-conformation syntaxin-independent effects of Munc18-1 in exocytosis. In order to test this hypothesis we expressed the Munc18-1 Glu379 insertion mutation in freshly cultured bovine adrenal chromaffin cells and assayed for a change in the number of dense-core granule release events by carbon fiber amperometry. Amperometry assesses release events by the oxidation of released neurotransmitter where individual oxidation spikes represent individual release events [34] and we have previously shown that expression of wildtype Munc18-1 has no effect on amperometric parameters in chromaffin cells [30]. Either control cells or cells expressing the Glu379 insertion were assayed for exocytosis in response to stimulation with a digitonin/calcium mix. Stimulation reliably produced amperometric responses from control cells, but only rarely from cells expressing the Glu379 insertional mutant (Figure 3A). Expression of the Glu379 insertion mutation caused a significant reduction in the total number of amperometric spikes per stimulus in comparison to controls in a direct comparison of responding cells (Figure 3B); however, it should be noted that this effect is likely underrepresented as the majority of Glu379 expressing cells produced no amperometric response.

**Figure 4:**
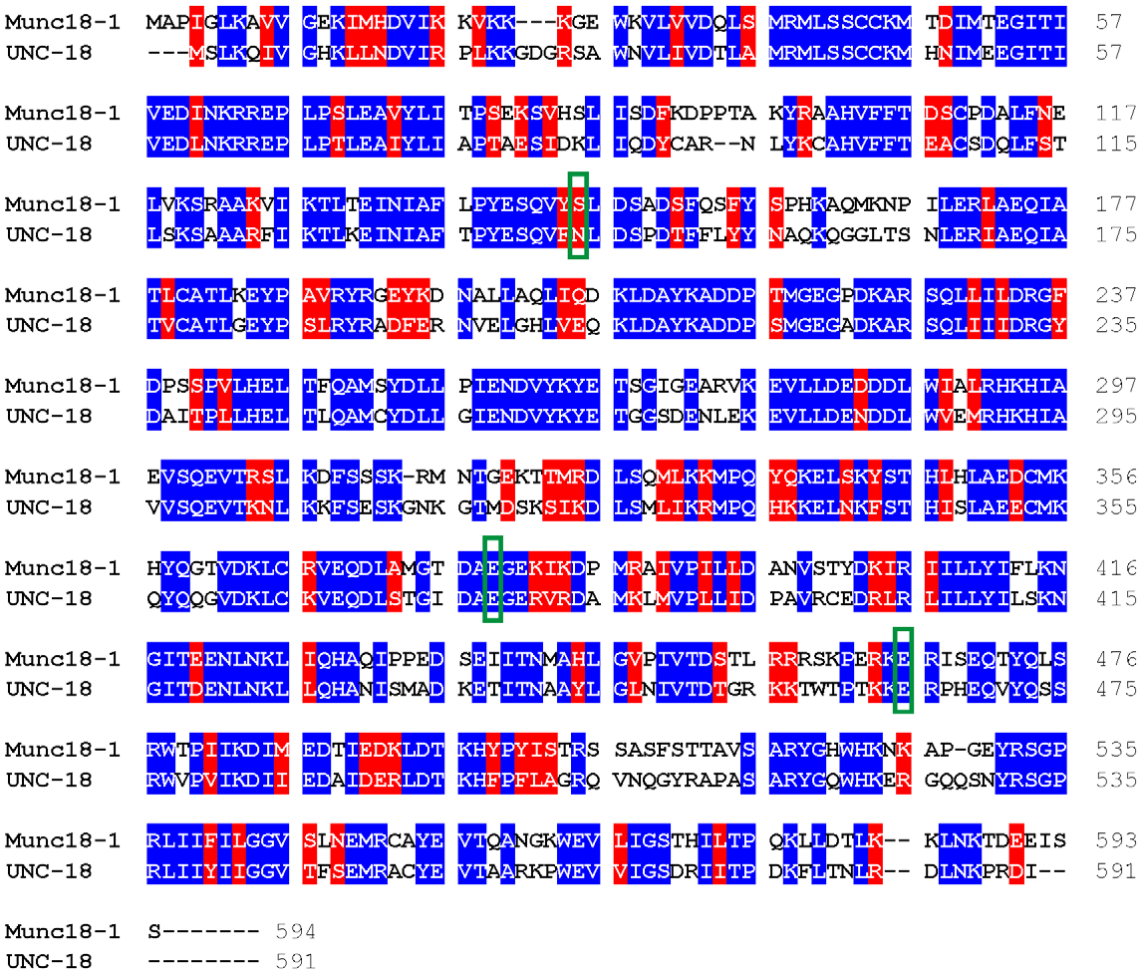
Sequence alignment of mammalian Munc18-1 and *Caenorhabditis elegans* UNC-18. The coding sequence of the two orthologous proteins were aligned. The positions of the two selected insertion mutants (Ser146 and Glu379 in Munc18-1; Asn144 and Glu378 in UNC-18) and the site of the point mutation (Glu466 in Munc18-1; Glu465 in UNC-18) are boxed in green. Identical amino acids are coloured blue; similar amino acids are coloured red.

**Figure 5:**
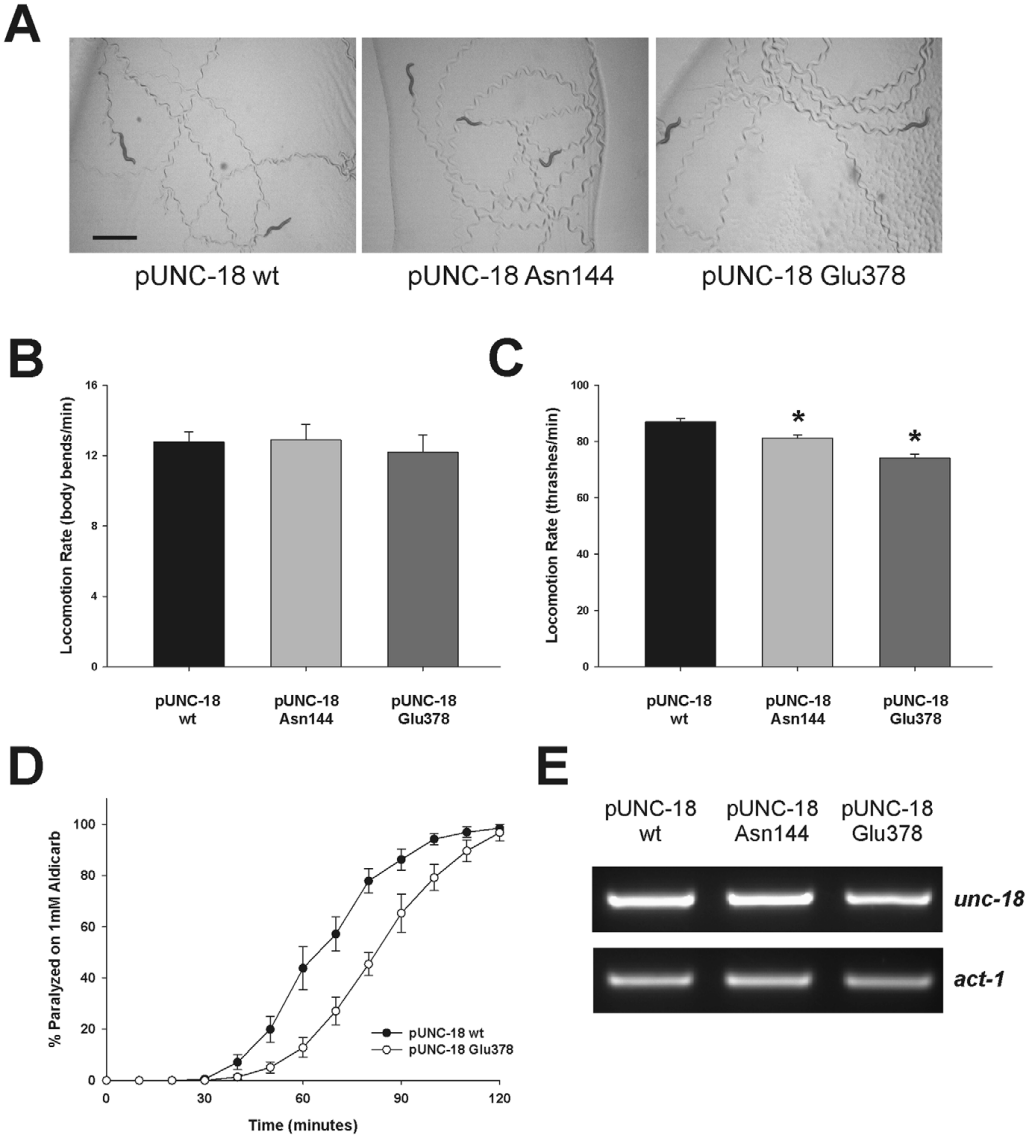
Transgenic *Caenorhabditis elegans* expressing the Glu378 insertion are resistant to the effects of aldicarb. (A) The *unc-18* (*e81*) null mutant was rescued with transgenic constructs expressing either wildtype (wt) UNC-18 or UNC-18 with the Asn144 or Glu378 insertion mutations. Photographs depict trails left in OP50 *E. coli* from 5 rescued worms after 5 minutes. Scale bar  = 1 mm. Locomotion rate for each transgenic rescue was then quantified by counting body bends per minute on unseeded solid agar (B) or by counting thrashes per minute in solution (C). There were no differences in locomotion rate between transgenic rescues on agar whereas both the Asn144 and Glu378 transgenic rescues moved slightly slower in solution (*; p<0.01). Data were analysed from 50 pUNC-18 wildtype, 46 pUNC-18 Asn144 and 25 pUNC-18 Glu378 worms (body bends) and 124 pUNC-18 wildtype, 94 pUNC-18 Asn144 and 126 pUNC-18 Glu378 (thrashes). (D) The Glu378 insertion mutant was resistant to the effects of aldicarb in comparison to wildtype (wt) rescues. Experiments were performed on a population of 20-25 adult hermaphrodite worms exposed to 1 mM aldicarb. Onset of paralysis was determined by mechanical stimulation every 10 minutes following exposure to the drug. Data were analysed from at least 3 separate experiments and represent the responses of multiple independently-derived transgenic rescues. (E) mRNA expression of *unc-18* was not different in transgenic rescues with wildtype (wt) *unc-18*, Asn144 or Glu378 mutants. Total RNA was purified from individual strains and used to generate cDNA. A similar level of amplification was achieved from all strains using plasmid-specific *unc-18* primers. Amplification of actin (*act-1*) was used as a control.

We further investigated the role of the Glu379 insertion mutation in exocytosis *in vivo* by creating transgenic *Caenorhabditis elegans* expressing UNC-18 with the equivalent mutational insert. SM proteins have very similar structures in general [9], [35], [36] and Munc18-1 and UNC-18 have a strong sequence conservation (Figure 4), thus allowing us to investigate the functional significance of the domain 3b insertion independent of Mint1 binding as worms do not possess a Mint orthologue that can bind UNC-18. We aligned the two coding sequences and created an identical insertion mutation (Table 1) at the orthologous Glu378 amino acid in UNC-18. *C. elegans unc-18* (*e81*) null worms were then rescued transgenically with an expression plasmid containing either wildtype *unc-18* or *unc-18* with the Glu378 or the Asn144 point mutation (equivalent to Munc18-1 Ser146). We have previously demonstrated phenotypic rescue of null worms with various constructs expressing *unc-18* under its endogenous promoter [16], [19]. We selected to use the Asn144 (Munc18-1 Ser146) as an additional control to wildtype *unc-18* as this insertion mutation had no apparent effects on binding in our assays. In comparison to transgenic rescues carrying the wildtype protein transgenic expression of either mutation rescued the *unc-18* (*e81*) paralytic phenotype, creating qualitatively normal worms (Figure 5A). We quantified the transgenic rescue of locomotion on solid agar (Figure 5B) and in solution (Figure 5C) and found that mutant worms locomoted statistically slower in solution in comparison to wildtypes, but were comparable to wildtype rescues on agar. This indicates that both insertion mutations could be expressed and neither produced massive detrimental effects on exocytosis. Cholinergic release at the worm neuromuscular junction was then more precisely assayed using the well-established aldicarb assay [37], [38]. Aldicarb, an acetylcholinesterase inhibitor, causes progressive paralysis in a population of worms and the rate of paralysis is indicative of the total amounts of neurotransmitter released at the neuromuscular junction. Mutants that reduce neurotransmitter release are resistant to the effects of aldicarb, whereas mutants that increase neurotransmitter release are hypersensitive. At 1mM aldicarb there was no effect of the Asn144 insertion in comparison to wildtype transgenic rescues as expected (Figure S1). In contrast, worms expressing the Glu378 insertion had a reduced sensitivity to aldicarb as demonstrated by the rightward shift in the worm population paralysis curve (Figure 5D). This indicates that the 5 amino acid insertion at Glu378 caused a decrease in the total amounts of acetylcholine release at the *C. elegans* neuromuscular junction. It is not anticipated that the effects seen here are due to differences in protein expression as identical phenotypes were exhibited by multiple independently-derived transgenic lines and analysis of mRNA expression revealed no obvious differences (Figure 5E).

**Figure 6:**
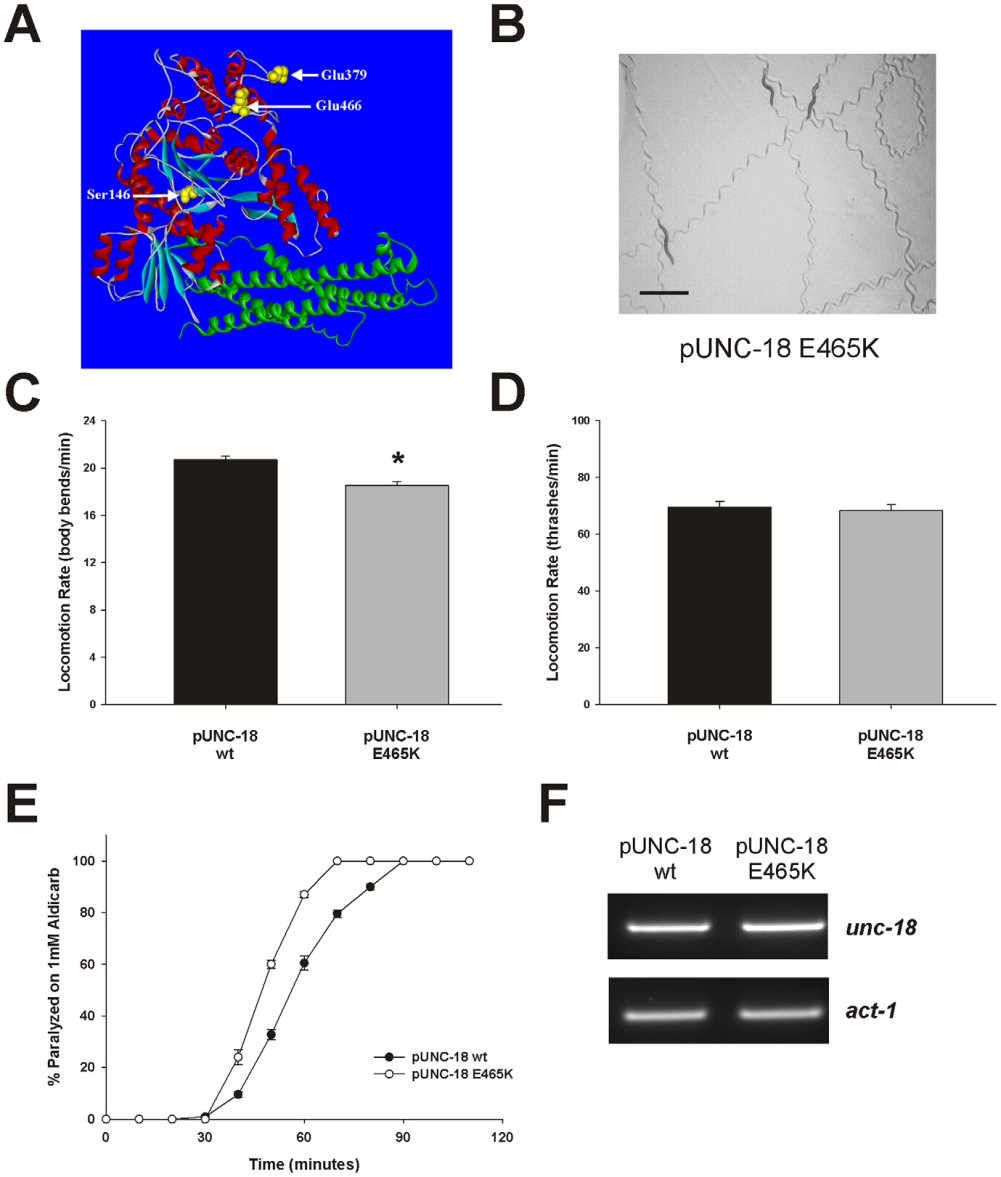
Transgenic *Caenorhabditis elegans* expressing the E465K UNC-18 point mutation are hypersensitive to the effects of aldicarb. (A) Structure of Munc18-1 protein indicating the position of the E466K point mutation in comparison to the Ser146 and Glu379 insertion mutations. (B) The *unc-18* (*e81*) null mutant was rescued with a transgenic construct expressing E465K UNC-18 (orthologous mutation to Munc18-1 E466K). The photograph depicts trails left in OP50 *E. coli* from 5 rescued worms after 5 minutes. Scale bar  = 1 mm. Locomotion rate for transgenic rescues was again quantified by counting body bends (C) or thrashing (D). There was a minimal reduction in locomotion rate as assessed by body bends (*; p<0.01); however, there was no significant difference as assessed by thrashing. Data were analysed from 20 pUNC-18 wildtype and 40 pUNC-18 E465K (body bends) and 81 pUNC-18 wildtype and 80 pUNC-18 E465K worms (thrashes). (E) The E465K point mutation worms were hypersensitive to the effects of aldicarb in comparison to wildtype (wt) rescues. Experiments were performed on a population of 20-25 adult hermaphrodite worms exposed to 1 mM aldicarb. Determination of paralysis and analysis were as described in the previous figure. (F) mRNA expression of *unc-18* was not different in transgenic rescues with wildtype (wt) *unc-18* or the E465K mutant. Total RNA was purified from individual strains and used to generate cDNA. A similar level of amplification was achieved from both strains using plasmid-specific *unc-18* primers. Amplification of actin (*act-1*) was used as a control.

The reduction in total neurotransmitter release indicated by the aldicarb assay results could be the result of a reduction in the total number of vesicle fusion events at the neuromuscular junction or a consequence of a large alteration in quantal size. Our amperometric results (Figure 3) pointed to an effect of the domain 3b insertion on the number of fusion events. We have previously tested various Munc18-1 mutations for alterations in exocytosis and found a number that affect quantal size and the kinetics of the fusion event [19], [29], [39], [40] but only one that altered the number of fusion events [29]. The E466K substitution causes an increase in the affinity of Munc18-1 for Rab3 and a concomitant increase in the number of single exocytotic events as assayed by carbon fiber amperometry [10], [29]. Glu466 is located in a linker region between domain 3b and domain 2 [9]; however, structurally the residue is relatively close to that of Glu379 (Figure 6a). We tested whether the previously observed increase in total exocytotic events as determined by carbon fiber amperometry would result in aldicarb hypersensitivity of *C. elegans*. To that end we transgenically rescued *e81* UNC-18 null mutants with constructs expressing UNC-18 E465K (orthologous to Munc18-1 E466K). As seen with the previous Glu378 and Asn144 insertions, expression of UNC-18 E465K created phenotypically normal worms with characteristic rates of locomotion both on agar plates and in solution (Figure 6B–D). We then compared these worms to transgenic worms rescued with wildtype protein in the 1mM aldicarb paralysis assay and found that they indeed had increased sensitivity to aldicarb as predicted (Figure 6E). Similar to the insertion transgenic worms, an analysis of mRNA expression revealed no obvious differences (Figure 6F). These data indicate that these mutations with altered numbers of vesicle fusion events in the mammalian chromaffin cell also have altered aldicarb sensitivity when expressed in *C. elegans*.

## Discussion

Previous work has demonstrated the importance of domains 1 and 3a of Munc18-1 in closed-conformation syntaxin binding [9] and specific point mutations in domain 1 can inhibit syntaxin binding [11], [29], [30]. In closed-conformation binding syntaxin is thought to reside within the arch-shape of Munc18-1, making connections to the inner face of the pocket [9]. Recent evidence suggests that Munc18-1 interacts with the assembled SNARE complex in a distinct fashion [41], [42] that may directly involve domain 3a [32], [43]. Alternative mutagenic studies using Sec1 have also pointed to domain 3a in SNARE complex binding [33] in accordance with a similar binding interface between Munc18-1 and either closed-conformation syntaxin or the assembled SNARE complex. Finally, binding between Munc18-1 and open syntaxin is thought to involve the extreme N-terminus of syntaxin [12], [13] and, again, domain 1 of Munc18-1 [12], [44]. Glu379 is located in domain 3b, structurally distant from any of the predicted syntaxin binding interfaces, and as such it is not surprising that mutational insertion here had no effect on measured closed-conformation syntaxin binding. Of the insertions that did affect syntaxin binding, Gln203, Leu410, Glu420 and Phe540 all likewise affected Mint binding (Figure 1). Although not thoroughly investigated here, it is possible that these insertions have affected whole protein conformation and thus potentially reflect nonspecific reductions in binding. Interestingly Leu410 and Glu420 both reside in domain 3b and affected Mint binding to a considerably greater extent than that of syntaxin. Leu118 surprisingly did not affect syntaxin binding; yet, this amino acid is structurally located within domain 1 and mutations in this region have been shown to affect binding to the N-terminus of syntaxin [16], [44]-[46]. The assay used for comparison of these mutants predominantly registers closed-conformation syntaxin binding; thus it is not expected that any potential alteration to the low-affinity N-terminal interaction [12], [14], [47] would be measurable here. We determined however that the Glu379 mutation did not affect Munc18-1 binding to the assembled SNARE complex (Figure 2C). We elected to use the Ser146 mutation as an insertion control as Ser146 is located outside of domain 1 and 3a and thus would not be predicted to affect syntaxin interactions (either to closed-conformation syntaxin or the N-terminus) and this was supported by our results as no effects on binding or exocytosis were demonstrated.

In addition to SNAREs Munc18-1 also associates with additional proteins at the synapse; however, very little experimental evidence exists for the structural domains of Munc18-1 involved in these interactions. Munc18-1 binds directly to granuphilin promoting vesicle docking [48], Doc2 binding interferes with the Munc18-1:syntaxin interaction [23], Rab3 binding enhances vesicle recruitment [10], interaction with phospholipase D inhibits its enzymatic activity [24] and binding to the Mint proteins potentially recruits Munc18-1 to neurexin:CASK complexes [49]. Despite experimental confirmation of these contacts only a handful of inhibitory point mutations in Munc18-1 for syntaxin-independent interactions have been characterised. The P242S mutation, originally isolated from *Drosophila* [50], reduces binding to Mint and alters the rate of exocytosis [29], [39]. Pro242 is located near the end of domain 2 of Munc18-1 structurally close to Glu379 and thus one could speculate that this exposed region of the protein is involved in Mint binding. The P242S mutation, however, has a completely different exocytotic phenotype [29] than the Glu379 insertion studied here and thus could be functionally distinct. The E466K mutation has no effect on Mint binding instead causing an enhanced affinity for Rab3 [10] and increasing the number of exocytotic events [29]. This mutation was originally modelled on the sly1–20 yeast mutation [51] which bypassed the requirement for the Rab protein Ypt1. This mutation is located in the linker region between domain 3b and 2 and is structurally adjacent to Glu379. We undertook a structural-functional approach to determine domains of Munc18-1 essential to closed-conformation syntaxin-independent function and, taken together, these data point to a novel role for domain 3b in transducing these interactions.

Functionally the results here point to domain 3b being involved with the recruitment/docking of vesicles. Munc18-1 has a docking phenotype in many systems including *C. elegans* [52], [53] and, although this docking function is thought to involve syntaxin binding, syntaxin-independent interactions may well contribute. In mammalian cells Munc18-1 E466K has an enhancement in recruitment/docking through an increased affinity for Rab3 [10] resulting in an increase in the number of fusion events [29]. The orthologous mutation investigated here caused a hypersensitivity to aldicarb in *C. elegans* (Figure 6) which is compatible with an increase in fusion events at the neuromuscular junction. Conversely, our Glu378 insertion mutant was resistant to aldicarb (Figure 5) consistent with the observed reduction in fusion events in chromaffin cells (Figure 3). Interestingly, *C. elegans rab-3* null mutants also display resistance to aldicarb with only mild locomotor defects [54]. Taken together our data point to a role for domain 3b in recruitment/docking of vesicles causing altered aldicarb sensitivity at the *C. elegans* neuromuscular junction. It is unlikely that the phenotypic effects of the insertion mutations are a result of potentially variable expression levels of the rescuing transgene as 3–5 independently-derived transgenic lines were generated for wildtype *unc-18* and each mutant. Phenotypic results were consistent for all lines whether they demonstrated significant effects (Figure 5, 6) or not (Figure S1) as has been previously demonstrated using other *unc-18* transgenics [19]. It is also unlikely that the effects of the domain 3b mutations are a function of a defect in syntaxin trafficking as this phenotype requires a complete absence of all modes of syntaxin binding which leave locomotion of transgenic worms severely impaired [16], [17]. Other interpretations cannot be ruled out however as many factors in vesicle priming have also been shown to alter aldicarb sensitivity in *C. elegans* such as UNC-13 [38] and Munc18-1 has established syntaxin-dependent roles in priming [20].

How might the functional role of domain 3b be conserved between mammalian Munc18-1, *C. elegans* UNC-18 and indeed other orthologous SM proteins such as yeast Sec1p? There is strong sequence similarity between Munc18-1 and UNC-18 (Figure 4) and this is certainly true for domain 3b (amino acids ∼362-456). There is considerably more divergence for other SM proteins although structurally the proteins are quite similar [9], [35], [36]. The positive correlation between mutations that affected neurotransmitter release in mammalian cells [29] and in *C. elegans* indicates these mutations have similar functional effects for both orthologous proteins. The Glu379 insertion was found to inhibit Mint1 binding, but this cannot be the explanation to its functional effects in *C. elegans* as UNC-18 does not bind the nematode Mint1 orthologue LIN-10 (J. Johnson, unpublished observations) as LIN-10 lacks the Munc18-1 binding domain (MBD) that is necessary for this interaction. It is possible however that there is an alternative protein in lower organisms that covers the functional role of SM protein – Mint interaction in exocytosis. For example, yeast also do not express Mint proteins but do express SM binding proteins such as Mso1p [55].

In conclusion, these data reveal a novel functional role for domain 3b of Munc18-1 in exocytosis. Recent work on Munc18-1 function has mostly taken the approach of introducing mutations based upon current structural information [16], [18], [20]. This process has been successful in identifying critical residues in domain 1 of Munc18-1 important for known specific protein interactions and their role in exocytosis. Here we have taken an unbiased genetic approach to identify functionally important domains of Munc18-1, utilising randomly inserted mutations in the Munc18-1 structure. Similar approaches have been used recently for Sec1p [33], [55] highlighting important domains involved in SNARE complex (domain 3a) and Mso1p binding respectively. The previous work on the significance of domain 3a has been supported by recent structural studies [32]. Further observations from unbiased experiments such as these could provide new alternative directions to the study of specific protein function by illuminating previously unknown domains of interest within Munc18-1/UNC-18.

## Materials and Methods

### Plasmid construction and recombinant protein production

For mammalian cell expression and transposon mutagenesis, Munc18-1 had been previously cloned into pcDNA3.1(-). Recombinant GST-tagged fusion proteins encoding Munc18-1, complexin, truncated (4-266) cytoplasmic syntaxin-1 and Mint1-Munc18 binding domain (MBD) were produced from pGEX-6p-1 vectors as previously described [29]. For use in fluorescence spectroscopy, the Munc18-1 Glu379 coding sequence in pcDNA3 was PCR amplified and the digested PCR product was ligated into the pGEX-6p-1 GST-fusion bacterial expression vector (GE Healthcare) by standard methods. GST-Munc18-1 or GST-Munc18-1 Glu379 plasmids were transformed into BL21 (DE3) *E. coli* and protein expression induced in parallel with 0.1 mM isopropyl-β-D-thiogalactopyranoside (IPTG) for 24 hrs at 18°C. His_6_-syntaxin1A4-266 protein was expressed in M15 (pREP4) *E.coli* for 3 hrs at 37°C with 1 mM IPTG. Proteins were purified from cytosolic fractions on glutathione-cellulose (Bioline, London, UK) or Talon (Takara BIO, France) affinity resins. GST-Munc18-1 proteins were treated with PreScission protease (40U, GE Healthcare) overnight at 4°C with constant agitation and GST-free protein collected in the supernatant fraction following centrifugation of glutathione-cellulose resin at 3,000 rpm for 1 min at 4°C. Eluted His_6_-syntaxin1A4-266 was dialysed extensively against binding buffer at 4°C to ensure removal of residual imidazole.

### Transposon mutagenesis of Munc18-1

To investigate domains of Munc18-1 important to exocytosis we randomly inserted small 15 bp linkers into the coding sequence. Random insertions in Munc18-1 were accomplished by a GPS-LS mutation reaction as per the manufacturer's instructions (New England Biolabs, MA, USA). Briefly, pcDNA-Munc18-1 plasmid was incubated with 1 µl transprimer donor DNA and 1 µl TnsABC transposase in 10X GPS buffer for 1 hr at 37°C. This reaction produces a series of plasmids with a small DNA cassette inserted randomly within the recipient plasmid. The reaction was stopped by heat inactivation and mutated plasmids were transformed into *E. coli* and incubated overnight at 37°C. Mutant plasmids were then screened by PCR for insertions specifically within the Munc18-1 open reading frame, whereas mutations introduced into the vector backbone were discarded. Mutant plasmids were isolated and the resultant DNA was digested with PmeI which removes the majority of the transprimer cassette leaving the 15 bp insertion. The 15 bp comprises an universal 10 bp insertion followed by a 5 bp duplication of the target DNA. Digestion products were separated on a 0.7% crystal violet gel, purified and religated back into pcDNA. This produced a pool of mutant Munc18-1 plasmids containing a 15 bp insertion.

### Binding of ^35^S-labelled *in vitro* transcription-translation-derived proteins to immobilised GST-fusion proteins

Following creation of Munc18-1 mutants with random 15 bp insertions ^35^S-labelled protein was produced using a T4 DNA polymerase TNT mix (Promega, WI, USA). Briefly, proportional volumes of TNT mix and [^35^S]methionine were incubated with mutant DNA at 30°C for 1.5 hours. Binding assays were performed using 96 well plates coated with glutathione as previously described [28]. GST, GST-syntaxin or GST-Mint1-MBD were added to the desired wells at a concentration of 200 ng/µl. Plates were then incubated at room temperature for 1 hour. After washing wells repeatedly with binding buffer (20 mM HEPES, 50 mM NaCl, 1 mM DTT, 2 mM MgCl_2_, 0.5% Triton X100, pH 7.4) 2.5 µl of Munc18-1 TNT-produced protein in binding buffer was added to each well and incubated at 4°C for 2 hours. Unbound protein was removed by multiple washes with binding buffer and bound protein was eluted in 100 µl elution buffer (10 mM glutathione, 50 mM Trizma-HCl, pH 7.4) at room temperature for 30 minutes. Eluted protein was added to scintillation vials containing 4 ml of cocktail T scintillant. The total amount of protein binding was assessed using a scintillation counter and results were recorded as counts per minute and compared as a percentage to binding of wildtype Munc18-1 protein. Analysis of binding of radiolabelled Munc18-1 to assembled SNARE complexes was carried out using a GST-complexin pulldown assay to isolate native SNARE complexes from a rat brain extract as described previously [33]. Data are shown as mean±S.E. Significance was tested by Student's t-Test.

### Fluorescence spectroscopy

Tryptophan fluorescence measurements were made on a JASCO FP-6300 spectrofluorometer instrument (JASCO, UK) using a 2.5 nm excitation band width and 10 nm emission band width. Briefly, Munc18-1 or Munc18-1 Glu379 proteins were prepared in binding buffer (50 mM Tris.HCl (pH 7.4), 150 mM NaCl) at a final concentration of 100 nM and transferred to a 1 ml quartz cuvette at room temperature prior to spectra acquisition. His_6_-syntaxin1A4-266 was subsequently added in 20 nM increments and new spectra acquired following brief mixing after each addition. Buffer alone and buffer plus maximal applied syntaxin concentration (200 nM) control spectra were also recorded. The Munc18-1 or Munc18-1 Glu379 alone peak fluorescence signal was set as 0% fluorescence shift and the maximum peak fluorescence signal observed at the highest concentration of syntaxin1A (200 nM) was set as 100% fluorescence shift. All other fluorescence shifts were normalised between these values and plotted as a function of syntaxin1A concentration. The data were fitted to Michaelis-Menton type kinetic plots by non-linear curve fitting using a hyperbolic function.

### Carbon fiber amperometry

Electrochemical recording of individual exocytotic events was performed as previously described [40], [56]. Freshly cultured bovine adrenal chromaffin cells (gift from Blacklidge Bros. Abattoir, Wigan, UK) were plated onto non-tissue culture treated 10 cm Petri dishes and left overnight at 37°C. Non-attached cells were then resuspended at a concentration of 1×10^7^ cells/ml in growth media. Plasmid containing Munc18-1 (Glu379) was mixed with a plasmid for enhanced green fluorescent protein (eGFP) and added to cells at 20 µg/ml. Cells were electroporated by a Bio-Rad Gene Pulser II (Bio-Rad, CA, USA), immediately diluted with fresh growth media to 1×10^6^ cells/ml and maintained in culture for 3-5 days. For carbon fiber amperometry experiments, cultured cells were first incubated in bath buffer (139 mM potassium glutamate, 0.2 mM EGTA, 20 mM PIPES, 2 mM ATP, 2 mM MgCl_2_, pH 6.6) and a 5 µm diameter carbon fiber was positioned in direct contact with a target cell. Exocytosis was initiated by pressure ejection of permeabilisation/stimulation buffer (139 mM potassium glutamate, 5 mM EGTA, 30 mM PIPES, 2 mM ATP, 2 mM MgCl_2_, 0.02 mM digitonin, 10 mM free calcium, pH 6.5) from a glass pipette situated on the opposite side of the cell. Amperometric responses were monitored using a VA-10 amplifier (NPI Electronic, Germany) and saved to computer by Axoscope 8 (Molecular Devices, CA, USA). Individual experiments were conducted in parallel from transfected and untransfected control cells from the same cell culture dishes. Transfected cells were identified by expression of GFP [40], [57]. Data were exported from Axoscope into Origin (Microcal Software, MA, USA) and spikes were selected for analysis if amplitude was greater than 40 pA. This amplitude cut-off was selected so that analyses represented exocytotic events arising from directly beneath the carbon fiber as small amperometric spikes can reflect events having originated from a site distant from the carbon fiber [58]. Data are shown as mean±S.E. Significance was tested by nonparametric Mann-Whitney U-test.

### Nematode culture, strains and microinjection

*C. elegans* were grown by standard methods on nematode growth media (NGM) at 20°C with *E. coli* OP50 as a food source [6]. This study used the *unc-18* (*e81*) strain which carries a point mutation causing a premature stop codon producing a paralytic null phenotype [59]. Germline transformation of *unc-18* (*e81*) worms with a plasmid construct carrying the *unc-18* cDNA under the control of its endogenous genomic flanking regions [60] was performed as previously described [16], [19]. Rescuing cDNA constructs (20 ng/µl) were coinjected with *sur-5*::GFP (20 ng/µl) in a total injected DNA concentration of 130 ng/µl. Successful rescue of the *e81* allele was determined by both rescue of phenotypically wildtype locomotion and GFP fluorescence. Point mutation (E465K) and transposon insertion were introduced into the *unc-18* cDNA using the Quikchange mutagenesis system (Stratagene, CA, USA).

### Reverse-transcription PCR

Animals were grown on two 60 mm NGM plates and harvested with chilled M9 buffer (42.3 mM Na_2_HPO_4_, 22 mM KH_2_PO_4_, 85.6 mM NaCl, 1 mM MgS0_4_). Worms were centrifuged at 5000 g for 5 minutes and the worm pellet was lysed in 400 µl TRI Reagent (Sigma). RNA was purified and first strand cDNA was produced from 1 µg total RNA template using AMV reverse transcriptase and random primers (Promega) following the manufacturer's instructions. PCR was performed with specific primers for *unc-18* using GoTaq DNA polymerase (Promega).

### Behavioural assays

The effects of the *unc-18* mutations on exocytosis were quantified in *C. elegans* by measurement of locomotion rate and aldicarb sensitivity. Locomotion rate was assessed by counting thrashes in 200 µl Dent's solution (140 mM NaCl, 6 mM KCl, 1 mM CaCl_2_, 1 mM MgCl_2_, 5 mM HEPES, pH 7.4, with bovine serum albumin at 0.1 mg ml^-1^) or body bends on fresh unseeded NGM plates [19], [61]. A single thrash or body bend was defined as one complete sinusoidal movement from maximum to minimum amplitude and back again. To correct for variation, young adult hermaphrodites were assayed for locomotion after the same amount of time in solution or on unseeded plates (10 minutes). Wildtype and mutant transgenic rescues were alternately measured to control for any small variation in environmental factors. For locomotion experiments at least 20 worms per strain were quantified. Acute sensitivity to 1 mM aldicarb (Sigma) was quantified by measuring the time to paralysis onset in a population of worms [19], [38]. For each experiment 20-25 worms were transferred to aldicarb plates and assessed every 10 minutes for paralysis after drug exposure by prodding with a thin tungsten wire. Experiments were performed at least three times. Data are expressed as mean±S.E and are representative of 3-5 independently-derived transgenic lines for each rescuing transgene.

## Supporting Information

Figure S1 **The UNC-18 Asn144 insertion mutation has no effect on aldicarb sensitivity.** The UNC-18 Asn144 insertion mutation transgenic worms were phenotypically identical to UNC-18 wt rescues with respect to aldicarb sensitivity. Experiments were performed on a population of 20-25 adult hermaphrodite worms exposed to 1 mM aldicarb. Determination of paralysis and analysis were as described in previous figures.
(TIF)
